# Spatial Disorientation in Alzheimer's Disease: The Missing Path From Virtual Reality to Real World

**DOI:** 10.3389/fnagi.2020.550514

**Published:** 2020-10-27

**Authors:** Vaisakh Puthusseryppady, Luke Emrich-Mills, Ellen Lowry, Martyn Patel, Michael Hornberger

**Affiliations:** ^1^Norwich Medical School, University of East Anglia, Norwich, United Kingdom; ^2^Research Development Programme, Norfolk and Suffolk National Health Service Foundation Trust, St Andrew's Lodge, Julian Hospital, Norwich, United Kingdom; ^3^Institute of Cognitive Neuroscience, University College London, London, United Kingdom; ^4^School of Psychology, University of East Anglia, Norwich, United Kingdom; ^5^Norfolk and Norwich University Hospitals National Health Service Foundation Trust, Norwich, United Kingdom

**Keywords:** spatial disorientation, spatial navigation, Alzheimer's disease, getting lost, virtual reality, real world

## Introduction

Spatial disorientation is one of the earliest symptoms in Alzheimer's disease (AD) (Coughlan et al., 2018), and has been increasingly measured using novel virtual reality (VR) paradigms in lab and clinical settings (Tu et al., 2015; Howett et al., 2019). At the same time, spatial disorientation often leads to AD patients getting lost in the real world (RW), with significant safeguarding and well-being implications (Alzheimer's Association., 2011; Rowe et al., 2015).

Overall, VR studies investigating spatial disorientation focus on underlying neurocognitive factors whilst RW studies highlight more external factors associated with this symptom. However, the link between the neurocognitive and external factors, and specifically how they might relate to each other has been relatively unexplored. We will highlight this gap in the literature by first giving an overview of VR studies of spatial disorientation in AD, before presenting evidence from RW studies of spatial disorientation in the community. Finally, we discuss the missing link between the VR and RW studies in more detail and how future research can overcome the limitations of the current literature.

## Virtual Reality Studies of Spatial Disorientation in AD

The advent of VR testing has led to a plethora of studies investigating spatial disorientation in virtual environments. The use of VR, either on a screen (i.e., non-immersive) or via an immersive head-mounted display, has offered many advantages for navigation testing. As VR navigation correlates highly with RW navigation (Coutrot et al., 2019), the technique/technology allows for controlled and systematic assessment of navigational abilities, offering a more ecologically valid alternative to standard visuospatial table-top tests (Lithfous et al., 2013; Mitolo et al., 2015). Fundamentally, these studies have been useful in highlighting how AD pathophysiology gives rise to impairments in the spatial navigation domain, particularly in the two main navigation strategies–egocentric and allocentric navigation.

Egocentric navigation is self-centered and involves encoding spatial representations of objects and locations in relation to the position of the navigator (Lester et al., 2017) (“*The shop is to the right of me”)*. This strategy is associated with a brain network centered around the parietal lobe and subcortical structures (Maguire, 1998; Latini-Corazzini et al., 2010). Conversely, allocentric navigation involves the use of non-self-centered cognitive maps, which contain encoded representations of spatial layouts and relationships from a survey-like perspective (Lithfous et al., 2013) (“*The shop is west of the town center”*). This strategy is associated with a brain network centered around the medial temporal lobe, particularly the hippocampus (Moffat et al., 2006; Lithfous et al., 2013). Everyday navigation requires a seamless integration of both egocentric and allocentric strategies, which is associated with activity in the retrosplenial cortex (Vann et al., 2009).

Several studies, including 11 using non-immersive and 2 using immersive VR, have reported AD patients to be impaired in egocentric and allocentric navigation, associated with pathology related changes to the medial temporal and parietal lobes (Hort et al., 2007; Jheng and Pai, 2009; Pengas et al., 2010; Nedelska et al., 2012; Vlcek and Laczo, 2014; Tu et al., 2015; Allison et al., 2016; Howett et al., 2019; Lowry et al., 2020). Some studies have additionally looked at the interaction between both navigation strategies. These studies report impairments in the translation/switching between strategies in AD patients (Pai and Yang, 2013; Serino and Riva, 2013; Serino et al., 2015) as well as AD patients adopting compensatory egocentric navigation strategies in response to compromised allocentric navigation with increasing dementia severity (Parizkova et al., 2018).

VR navigation studies have also explored how landmark recognition, critical to both egocentric and allocentric navigation, is altered in AD (O'Malley et al., 2017). These studies report deficits in landmark recognition (Zakzanis et al., 2009) as well as impairment on tests of landmark identity, recall, location, temporal order, directional knowledge, and scene recognition in AD patients (Allison et al., 2016).

## Real World Studies of Spatial Disorientation in AD

Compared to VR, RW studies on spatial disorientation in AD are limited. This is largely due to the relative lack of experimental control over contextual factors associated with RW settings (i.e., changing patterns of weather, crowds, noise etc.). This lack of control makes it challenging to keep environments consistent over time for repeated navigation testing (Davis and Ohman, 2016). Moreover, RW navigation tests are considered impractical to administer clinically due to different RW settings, thereby making comparison across sites challenging (Pengas et al., 2010). Nevertheless, with spatial disorientation often causing patients to get lost in the community (Rowe et al., 2011), much of the RW studies have focused on examining factors that contribute to these missing incidents.

The majority of RW studies have focused on key contextual factors contributing to missing incidents in AD patients. In particular, temporary gaps in patient supervision by the caregiver, such as when the patient performs a routine activity (i.e., neighborhood walks), when they are temporarily left alone on purpose, or during the night when the caregiver is sleeping are key contextual factors (Rowe et al., 2015). These findings have been complemented by studies investigating demographic and environmental risk factors for missing incidents. A common finding across studies is that more patients go missing from domestic residences when compared to care settings (Rowe et al., 2011; White and Montgomery, 2015; MacAndrew et al., 2018; Puthusseryppady et al., 2019). Additionally, higher age, longer duration of time missing, and cooler months have been reported as potential risk factors for lost patients sustaining harm (White and Montgomery, 2015; Lissemore et al., 2019). Finally, increased outdoor landmark density has been suggested as an environmental risk factor for missing incidents (Puthusseryppady et al., 2019).

On a neurocognitive level, RW studies have suggested that impairments in various cognitive processes can contribute to AD patients going missing including topographical memory, object recognition, as well as the modulation of visuospatial processing by working memory and executive functions (Guariglia and Nitrini, 2009; White and Montgomery, 2014; Yatawara et al., 2017). For spatial navigation measures, to our knowledge only questionnaire based information has been used to predict the incidence of getting lost for patients (Pai and Lee, 2016) While younger age was reported as being a predictor of getting lost, the presence of a safety range (i.e., restricting navigation to very familiar places) was found to be a protector for getting lost recurrence in patients (Kwok et al., 2010).

Beyond missing incidents, some studies have investigated the navigation of AD patients in controlled and naturalistic RW environments. In controlled environments (i.e., hospital settings, floor mazes), studies show that AD patients exhibit impairments in egocentric navigation, which was associated with decreased volumes of the right posterior hippocampus/parietal cortex, landmark recognition/recall, and allocentric processes (Cherrier et al., 2001; DeIpolyi et al., 2007; Benke et al., 2014; Tangen et al., 2015; Zanco et al., 2018). Meanwhile, in naturalistic environments (i.e., familiar neighborhoods) studies report that in familiar settings, AD patients increasingly use visible landmarks as navigation aids and are more likely to exhibit spatial disorientation/get lost when compared to controls (Sheehan et al., 2006; Olsson et al., 2019).

## Discussion

Taken together, spatial disorientation in AD has mainly been explored through VR rather than RW approaches. Although VR environments have provided useful insight into how navigation strategies are affected in AD and are increasingly being used to test these abilities in patients at different disease stages, there are some limitations associated with this approach.

Practically, it can be challenging for elderly AD patients to perform VR tasks on the computer and VR-induced motion sickness remains a relevant concern in this population (Bohil et al., 2011; Verghese et al., 2017). Similarly, VR navigation may not capture the vividness of RW settings since they often lack auditory/olfactory cues or locomotion, a crucial feature of RW navigation (van der Ham et al., 2015; O'Malley et al., 2017; although see Howett et al., 2019 for a VR paradigm incorporating RW walking). Most importantly however, despite VR navigation tasks showing sensitivity and specificity in engaging the brain navigation systems, they often do not represent the daily navigation challenges faced by AD patients that lead to spatial disorientation in the RW. Hence, the utility of current VR studies in understanding why and how spatial disorientation manifests in the RW for AD patients is unclear.

**Figure 1:**
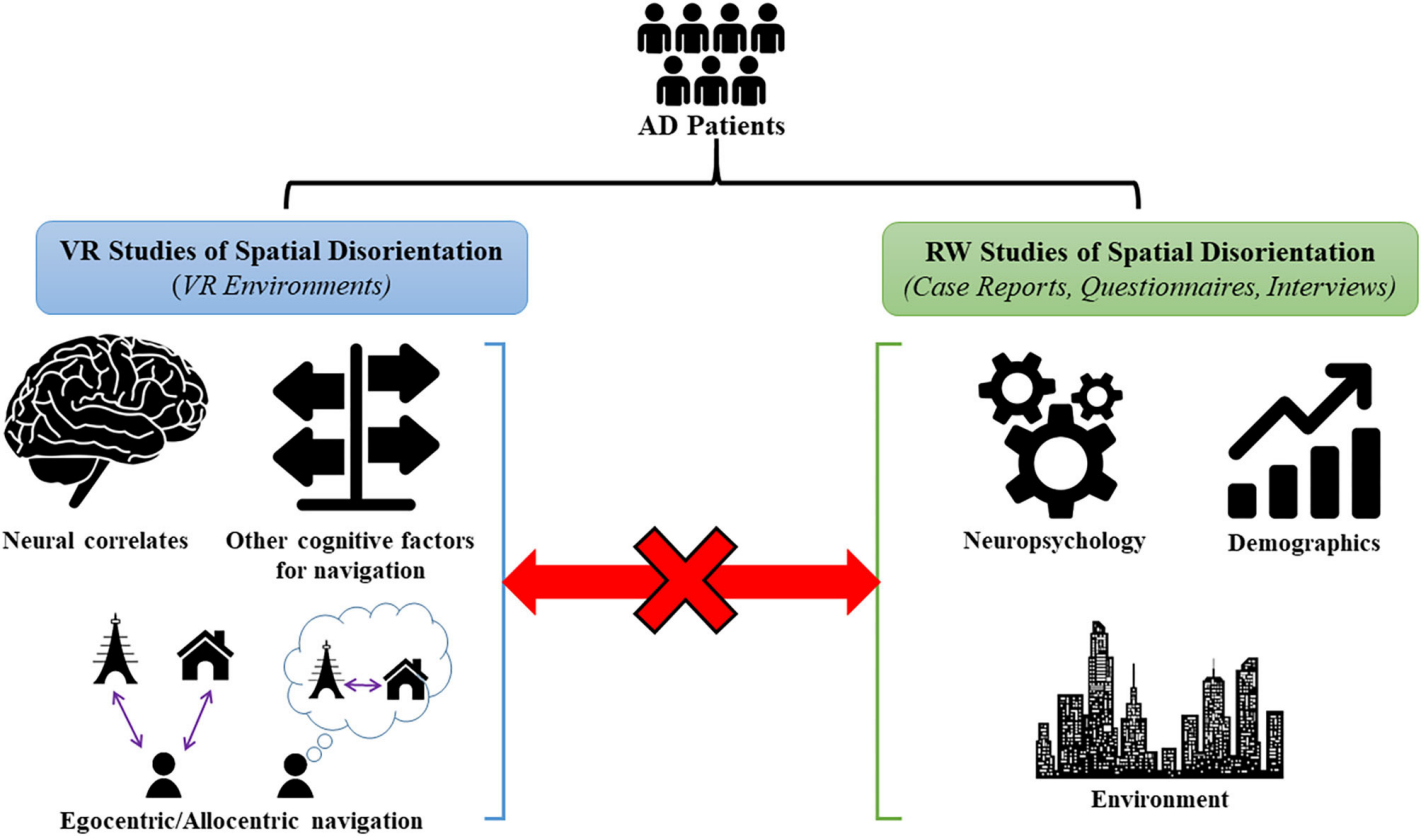
Summary of VR and RW studies of spatial disorientation in AD. VR studies have utilized VR environments to identify the underlying neural correlates of spatial navigation, impairments in egocentric/allocentric navigation, and in other cognitive factors used in navigation (visuospatial memory, episodic memory, and attention for landmarks etc.) in AD patients. RW studies have mainly studied missing AD patients in the community using case reports, questionnaires and interviews, and have identified neuropsychological, demographic, and environmental risk factors for these incidences. At present, there is a missing link between the VR and RW studies as no studies have specifically related the RW factors to underlying impairments in the spatial navigation domain^1^.

Conversely, the RW studies have focused more on external factors (contextual, demographic, environmental) associated with missing incidents in AD patients. However, most RW studies do not link to the underlying spatial navigation brain changes or pathophysiology in AD. Although some studies attempted to relate the external factors to neurocognitive findings in patients, these findings were mostly based on neuropsychological tests which do not measure spatial navigation *per se* but more generally visuospatial impairment. In addition, despite few studies relating RW spatial disorientation to the underlying navigation processes, these studies mostly used unfamiliar, controlled environments and hence suffer from the same limitations of VR studies in not accurately capturing common situations where patients experience spatial disorientation. Therefore, it is currently unclear whether RW spatial disorientation in AD may be caused by compromised spatial navigation or more general cognitive deficits (eg. executive function and visuospatial impairments) (Rowe et al., 2012). Based on this, there is a missing link between VR and RW studies on spatial disorientation in AD (Figure 1).

With current VR and RW studies shedding light on neurocognitive/external factors, respectively, an approach combining VR with RW navigation would offer the best chance to study interactions between these types of factors and investigate how their alterations could lead to spatial disorientation in AD. We suggest a potential experimental model using navigation tests in naturalistic RW settings, like familiar neighborhoods used in previous studies (Sheehan et al., 2006; Olsson et al., 2019), to assess how patients use egocentric/allocentric navigation on a daily basis and to more accurately simulate situations where patients may experience disorientation. Here, it would be useful to more closely study external factors influencing navigation (e.g., outdoor landmarks, road network structure, visibility, etc.) in locations where patients feel disoriented, to identify environmental risk factors for spatial disorientation. In particular, VR elements could be introduced into the RW settings via augmented reality displays to study how changing relevant environmental features influence patients' navigation/disorientation behavior. Additionally, tracking patients' outdoor movement using sensor devices (e.g., GPS tracking) allows the use of machine learning approaches to detect more subtle disorientation behavior patterns and how this varies according to navigation strategy use/environmental features. Overall, the RW component of our model can offer insight into whether spatial disorientation occurs when patients are unable to use a specific navigation strategy in certain types of environments. On the other hand, classic VR environments can also be used separately from the RW to test patients' navigation abilities more systematically. These results can then be related to patient performance on the RW tests to investigate whether one can predict patients at a high risk of exhibiting spatial disorientation in the RW based on VR alone. In line with our VR-RW model are successful approaches used by recent healthy aging studies including a realistic VR version of a RW town, immersive VR with RW walking, and VR navigation to predict navigation in complex RW environments (Chen et al., 2017; Coutrot et al., 2019; Hilton et al., 2019).

In conclusion, in our opinion it is important that future studies relate VR navigation results to RW factors to gain a more holistic view of factors contributing to spatial disorientation in AD. Theoretically, this would allow greater ecological validity of VR tasks and might inform future VR task designs. Clinically, this approach could help enhance our understanding of getting lost events in AD patients, which in turn would allow use of VR for predicting patients at higher risk for these events before they actually occur. This could then be used to inform and implement much-needed, effective safeguarding strategies to prevent AD patients from getting lost in future, which currently are very limited (Emrich-Mills et al., 2019).

## Author Contributions

VP and MH designed the manuscript. VP, LE-M, EL, MP, and MH wrote the manuscript. All authors contributed to the article and approved the submitted version.

## Conflict of Interest

The authors declare that the research was conducted in the absence of any commercial or financial relationships that could be construed as a potential conflict of interest. The reviewer LS declared a past co-authorship with one of the authors MH to the handling editor.

## Footnotes

^1^Icons used in the figure – “People” icon by Alice Design, “Brain” icon by Clockwise, “Gears” icon by Daniel Shoreman, “Person” icon by Support Designs, “House” icon by David, “Tower” icon by ibrandify, “Navigation” icon by Jejen Juliansyah Nur Agung, “City” icon by sumhi_icon, and “Statistics” icon by Adrien Coquet, all from thenounproject.com.
